# Mitochondria-targeted therapy with metformin and MitoQ reduces oxidative stress, improves mitochondrial function, and restores metabolic homeostasis in a murine model of Gulf War Illness

**DOI:** 10.1016/j.redox.2025.103714

**Published:** 2025-06-04

**Authors:** Lun Cai, Mundanattu Swetha, Abraham Raji, Alvin V. Terry, Raghavan Pillai Raju

**Affiliations:** a Department of Pharmacology and Toxicology, Medical College of Georgia, Augusta University, Augusta, GA, USA; b Charlie Norwood Veterans Affairs Medical Center, Augusta, GA, USA

**Keywords:** Gulf War Illness, Metformin, MitoQ, AMPK, Mitochondria

## Abstract

Gulf War Illness (GWI) is a cluster of medically unexplained chronic symptoms, including neurological and gastrointestinal impairments, and muscle fatigue, suffered by veterans of the Persian Gulf War. A GWI model in C57BL/6 mice exposed to the nerve gas prophylactic pyridostigmine-bromide (PB) and the insecticide permethrin (PER) was used to test the effect of mitochondria-potentiating agents, metformin and MitoQ on chronic fatigue, observed in GWI. The exposure of mice to PB/PER resulted in enhanced oxidative stress, impaired mitochondrial function, and reduced autophagy. Treatment with the anti-diabetic drug metformin and the mitochondria-targeted antioxidant available as a dietary supplement, MitoQ, activated the AMPK signaling, reduced oxidative stress, and attenuated inflammation in gastrocnemius muscle tissue compared to untreated mice. The combination of metformin and MitoQ was found to be more effective than the individual treatments in activating AMPK. The combination treatment rescued autophagy and improved mitochondrial respiration. Chronic fatigue assessments by the hanging wire and rotarod tests, and voluntary wheel running activities showed improved physical activity/strength in mice treated with metformin and MitoQ. The results suggest the potential therapeutic benefit of a combination formulation of metformin and MitoQ in addressing the molecular and energetic impairments of skeletal muscle in GWI.

## Highlights

• In a mouse model of GWI, Metformin and MitoQ treatment improved behavioral signs of GWI.
• Metformin and MitoQ reduced tissue inflammation in the skeletal muscle of mice with GWI.
• Metformin and MitoQ treatment ameliorated GWI-induced oxidative stress and improved autophagy.
• Metformin and MitoQ improved the GWI-induced decrease in cellular respiration in the gastrocnemius muscle.

## Graphical abstract

**Figure:**
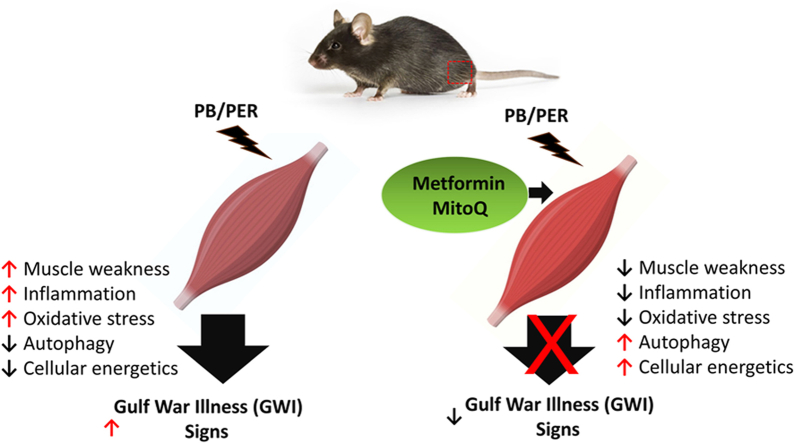

## 1 Introduction

Approximately one-third of the veterans of US and coalition forces from the Persian Gulf War after Operations Desert Shield and Desert Storm reported a variety of adverse health effects [1]. Many of the cases include combinations of nonspecific symptoms such as neurological and gastrointestinal distress, musculoskeletal pain, chronic fatigue, respiratory difficulties, headaches, mood disorders, cognitive impairments and insomnia. These clusters of medically unexplained chronic symptoms, suffered by veterans of the Gulf War are known as Gulf War Illness (GWI) [2]. The United States Centers for Disease Control and Prevention (CDC) defines GWI as a multi-symptom illness having at least one symptom like fatigue, mood-cognition, and musculoskeletal problems from a cluster of symptoms persistent for more than 6 months [3].

The etiology of GWI has been hypothesized to include many factors such as chemical, physiological, and environmental stressors present in the war zone. It is estimated that soldiers were exposed to exceptionally large amounts of more than 35 different types of pesticides and insecticides, including toxic organophosphates such as azamethiphos, chlorpyrifos, diazinon, dichlorvos, malathion and carbamates such as propoxur, carbaryl, and methomyl, pyrethroids such as permethrin (PER) and fumes and smoke from military operations, oil well fires, diesel exhaust, toxic paints, sand, depleted uranium, infectious agents, chemoprophylactic agents, multiple immunizations and the nerve gas prophylactic pyridostigmine bromide (PB), which is a quaternary carbamate inhibitor of acetylcholinesterase. Studies have shown that PB and PER are among the two potential chemicals contributing to GWI [[4], [5], [6], [7]]. PB is an oral medication for myasthenia gravis, that was used as a pretreatment against potential Iraqi attacks with nerve gas agents. Nerve gas agents such as soman act by irreversibly binding and inhibiting acetylcholinesterase, this results in excessive accumulation of acetylcholine in the synapse. PB is a reversible inhibitor of acetylcholinesterase thus blocking nerve gases from permanently inactivating acetylcholinesterase [8]. PER is a synthetic pyrethroid insecticide topically applied on the Gulf War personnel and their clothes, which binds to the voltage-gated sodium channels (VGSCs) leading to prolonged opening and alterations in neuronal firing and thereby killing the insects [9].

GWI has been persistent in veterans, even 30 years after the Gulf War, due to a lack of effective treatments. The research over the past few years in GWI veterans revealed that an increase in inflammatory cytokine production and oxidative stress together may contribute to a vicious cycle of mitochondrial dysfunction and energetic failure to augment ROS production and trigger further progressively increasing mitochondrial stress [[10], [11], [12]]. Therefore, we hypothesized that agents capable of improving mitochondrial function have the potential to restore cellular energetics and treat GWI. Metformin, a first-line antidiabetic drug taken by over 150 million people annually [13] was reported to activate AMPK and improve mitochondrial respiration [14]. Mitoquinone (MitoQ; 10-(4,5-dimethoxy-2-methyl-3,6-dioxo-1,4-cyclohexadien-1-yl)decyl triphenylphosphonium) is a mitochondria-targeted molecule specifically designed to decrease mitochondrial oxidative stress that has been evaluated in clinical trials [15,16]. To test our hypothesis, we used an established GWI mouse model [[17], [18], [19], [20]] and evaluated the effect of the two mitochondria-targeting agents, metformin and MitoQ, individually and in combination.

## 2 Materials and Methods

### 2.1 Animals

The experiments conducted on the mice were approved by the Augusta University Institutional Animal Care and Use Committee as per the guidelines of the National Institute of Health and approved by the appropriate DOD regulatory agency (ACURO). Wild-Type (WT) C57BL/6 mice were purchased from Jackson Laboratories and allowed to acclimatize to the animal facility for 2–4 weeks before use. The animals were housed in the vivarium at 18-23°C with 40–60% humidity, free access to food and water, and 12-h light/dark cycles. Male mice were chosen for the study because most of the service personnel participated in the Gulf War were males.

### 2.2 Development of GWI model in mice

**Fig. 1:**
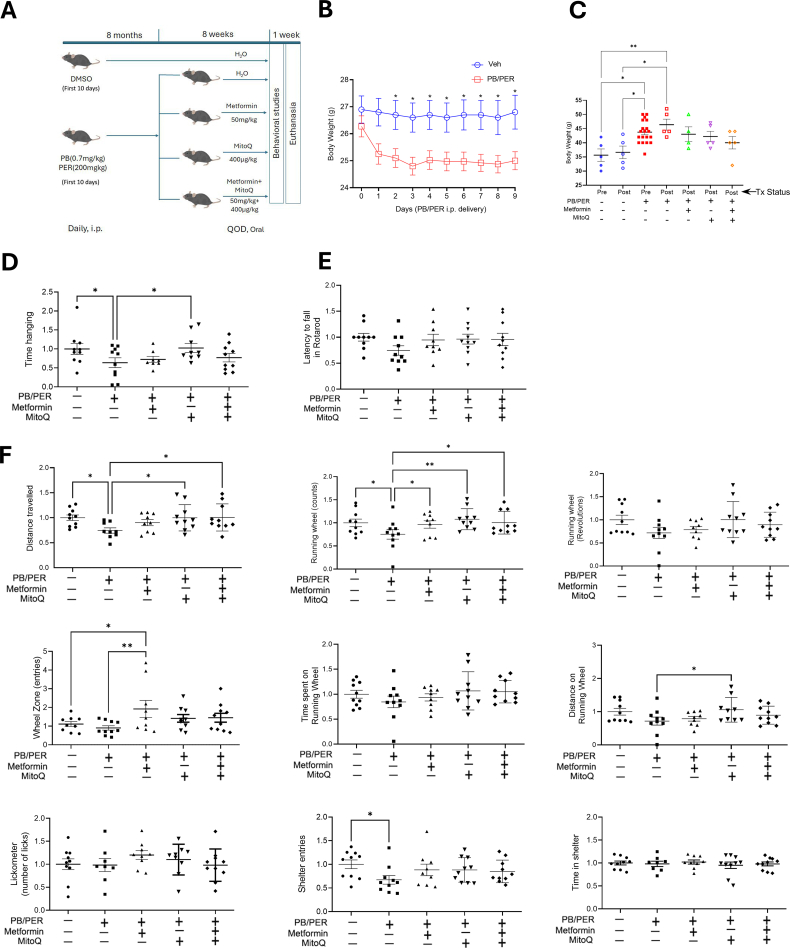
**The effect of** m**etformin and MitoQ on behavioral signs in mice exposed to PB/PER. (A)** Schematic representation of Gulf War Illness study using C57BL/6 mice **(B)** Body weight of mice during PB/PER injection period, n = 49 (10 and 39); vehicle (Veh) = DMSO. Multiple *t*-test used for p value; ∗p < 0.05 **(C)** Body weight of mice before (at the end of 8 months post-PB/PER exposure) and after the 8-week treatment (oral) with vehicle (distilled water), metformin and/or MitoQ. The treatment was for 2 months. Tx = treatment with vehicle, metformin and/or MitoQ. One way ANOVA was used to calculate significance. The graphs are shown as individual data points along with Mean ± SEM. n = 4-19. **(D**–**F)** Behavior analysis **(D)** Hanging wire test **(E)** Rotarod test, and **(F)** Behavior tests using Noldus Phenotyper. See the original data in Table S2. The experiment was conducted in two batches, with all groups tested in each batch. The graphs are shown as individual data points along with Mean ± SEM. n = 8–10, One-way ANOVA was used for analysis. ∗p < 0.05,∗∗p < 0.01.

10–12 weeks old male C57BL/6 mice received freshly prepared 0.7 mg/kg of PB and 200 mg/kg of PER (GWI agents) dissolved in dimethyl sulfoxide (DMSO, 100%) daily for ten consecutive days, intraperitoneally, while the control mice received an equal volume of DMSO (50 μl). Eight months after exposure to GWI agents, the animals were treated with 50 mg/kg of metformin, 400 μg/kg of MitoQ, a combination of both drugs, or vehicle (PB/PER group) by oral gavage on alternate days for 8 weeks (Fig. 1A). The duration and dose of treatment with GWI agents and the later drug treatment for 8 weeks were previously standardized by other groups [18,19,]. The PB/PER doses were based upon average body weight, the drug usage, and the LD50. Though a latency period of 5 months after the PB/PER treatment, until the beginning of the drug treatment, was used by others, we found that the 8-month latency period resulted in a more marked change in the behavior parameters tested. The behavioral studies were carried out after the treatment period, the animals were sacrificed, and tissues collected for molecular analysis.

### 2.3 Behavioral studies

Behavioral studies were conducted at the Small Animal Behavioral Core (SABC) at Augusta University immediately after the treatment regimen.

#### 2.3.1 Hanging wire test

The hanging wire test was used to determine any muscle dysfunction or neuromuscular deficiencies in mice administered with PB/PER (GWI mice) and treated with or without metformin/MitoQ. In the test, mice were acclimated to the testing room for 30 min and gently placed on a wire grid top (1.3 cm grid mesh) with the top inverted and suspended above a 42 x 42 × 30 cm acrylic box filled with standard corncob bedding. Each mouse was given one 5-min training trial where they were repeatedly placed back on the wire grid after falling off to reinforce the mouse to stay on the grid. Following the training trial, each mouse was given 3 trials with an inter-trial interval of 15 min and latency to fall (sec) recorded.

#### 2.3.2 Rotarod methods

Mice were acclimated in the lab for 30 min before the rotarod assessment. Each mouse was placed on an individual stationary rod, and the rod rotated after 10 s. The rod gradually increased in speed from 0 to 10 rpms within a 1 min interval. The duration of a single trial was 5 min with an inter-trial interval of 20 min. Each mouse was given 3 separate training trials on day 1. Twenty-four hours after training, animals were returned to the lab for 30 min acclimation followed by rotarod assessment at 0–23 rpms. Initially, all animals were given a “refresher” trial at 0–10 rpms within 1 min to ensure stable performance. During the test session, animals were given 3 separate trials 5 min in length with an inter-trial interval of 20 min. Each animal was placed on the stationary rod and after 10 s the speed increased from 0 to 23 rpms within a 2 min interval (final speed reached by 2 min). Latency to fall was recorded for each trial and an average (±S.E.M.) calculated.

#### 2.3.3 Phenotyper assessments

The Noldus PhenoTyper® was used to monitor general home cage activity. Each mouse was placed in an individual PhenoTyper® chamber (30×30×35cm). Each chamber had a running wheel, an IR translucent shelter, food freely available in a hopper and water delivered by a lickometer connected to a water bottle. The floor was covered with 3 cm standard corncob bedding. Each chamber was fitted with an IR camera system to allow for monitoring in complete darkness. Subjects were acclimated to the boxes in an 18-h habituation phase starting around 4 p.m. and monitoring through the animal's dark cycle. One day later, the subjects were returned to the chambers for the test phase and were monitored as performed for the habituation phase, beginning around 4 p.m. for 18 h. Measures included running wheel activity (distance, time, and frequency), number of licks for water consumption and time and frequency in the shelter.

### 2.4 Real-time quantitative reverse transcription PCR (RT-qPCR)

Total RNA was extracted from the gastrocnemius muscle tissues of all groups of mice using Trizol-reagent according to the manufacturer's instructions (Invitrogen, Carlsbad, CA, USA) [21,22]. The RNA quality was ascertained by A260/280 ratio. 1 μg of total RNA was reverse transcribed to cDNA using ImProm-II reverse transcription system (Promega, Madison, WI, USA). Gene-specific primers were synthesized from Invitrogen (Thermo Fisher Scientific, Inc., Carlsbad, CA, USA) (Table S1). The reverse-transcribed transcripts were analyzed using Bio-Rad iTaq Universal SYBR Green Master Mix (Bio-Rad, Hercules, CA, USA) in areal-time PCR machine (Agilent Technologies, Santa Clara, CA, USA). The expression levels of the genes were normalized to those of β-actin. 2^−ΔΔCt^ values were used for statistical analyses.

### 2.5 Biochemical assays

#### 2.5.1 ATP assay

The quantitative determination of ATP was performed in 96-well plates using the ATP Assay Kit (ab83355, Abcam, Waltham, MA, USA)with colorimetric analysis. The assay employed recombinant firefly luciferase and its substrate D-luciferin. Briefly, the reaction mixture containing luciferase and luciferin was prepared, and the background luminescence was measured. ATP standards were used to confirm linearity and luminescence. ATP concentration was deduced from the standard curve as nmol/well and normalized to total protein.

#### 2.5.2 Citrate assay

Citrate levels in gastrocnemius tissues were measured using Citrate assay kit (MAK333, Sigma-Aldrich, MO, USA) according to the manufacturer's protocols. The samples were tested in duplicates.

#### 2.5.3 MDA assay

Malondialdehyde (MDA) levels were measured using TBARS (TCA Method) assay Kit (700870, Cayman Chemical, Ann Arbor, MI, USA). Briefly, the gastrocnemius tissue homogenates together with TCA assay reagent forms MDA-TBA adduct under high temperature and acidic conditions and is measured colorimetrically at 530–540 nm.

#### 2.5.4 MPO assay

Myeloperoxidase (MPO) activity was measured using a MPO activity assay kit (ab111749, Abcam, Waltham, MA, USA) in alignment with the manufacturer's instructions. Briefly, snap-frozen gastrocnemius tissues (10 mg) were homogenized in 100 μL of cold MPO buffer and centrifuged (15,000×*g* for 15 min at 4 °C), supernatants were collected and incubated using MPO reaction mix. The fluorescence signal was read at Ex/Em = 484/525 nm. MPO activity was normalized to total protein concentration.

#### 2.5.5 NAD/NADH assay

NAD/NADH levels in mice gastrocnemius tissues were measured using a commercially available kit (MAK037, Sigma Chemical, St. Louis, MO, USA) according to the manufacturer's instructions. NAD total (NAD and NADH) or NADH levels were quantified in a colorimetric assay at 450 nm using Epoch Microplate Absorbance Reader (Agilent, Santa Clara, CA, USA).

#### 2.5.6 PDH enzyme activity

PDH enzyme activity of gastrocnemius tissues were measured at 450 nm using a colorimetric PDH Enzyme Activity Kit (ab109902, Abcam, Cambridge, MA, USA) according to the manufacturer's directions. Briefly, 100 μg of protein with reaction mix was added to the PDH enzyme immunocaptured within the wells of the microplate included with the kit. Activity was determined by following the reduction of NAD+ to NADH, coupled with the reduction of a reporter dye to yield a colored reaction product with an increase in absorbance at 450 nm at RT.

### 2.6 Western blotting analysis

The gastrocnemius tissues were lysed in RIPA buffer (Thermo Scientific, Chicago, IL, USA) containing 25 mmol/L Tris-HCl pH 7.6, 150 mmol/L NaCl, 1 % NP-40, 1 % sodium deoxycholate, 0.1 % SDS and Pierce protease inhibitor cocktail The tissue lysates were centrifuged at 14,000 g for 10 min and the supernatant saved for protein estimation and analysis. Protein aliquots were combined with 4X Loading dye and resolved in SDS polyacrylamide gel, transferred to PVDF membrane, blocked using 5 % (w/v) non-fat dried milk in Tris-buffered saline containing 25 mmol/L Tris-HCl (pH 7. 4), 0.13 mol/L NaCl, 0.0027 mol/L KCl and 0.1 % Tween 20 for 1 h at room temperature (RT) and then incubated with respective antibodies overnight at 4 °C. The primary antibodies used were: *p*-AMPKα (Cell signaling, 50081), AMPKα (Cell signaling, 5831), *p*-ACC (Cell signaling, 11818), ACC (Cell signaling, 3676), *p*-ULK1(Cell signaling, 89267), ULK1(Cell signaling, 8054), p62 (Abcam, ab 56416), *p*-Beclin 1 (Cell signaling, 54101), Beclin 1(Cell signaling, 3738), LC3B (Cell signaling, 2775), Nrf2 (Cell signaling, 20733), BACH1 (Santa Cruz, sc-271211), HO-1 (Cell signaling, 43966), VDAC1 (Abcam, ab15895), OXPHOS (Abcam, ab110413), Sirt-1 (Cell signaling, 9475), Pgc-1α (Abcam, ab54481) and β-actin (Abcam, ab8227). The membranes were subsequently washed and incubated with horseradish peroxidase-conjugated secondary antibody for 1 h at RT and developed using Clarity Western ECL substrate (BioRad). Protein bands were developed using ChemiDoc (Bio-Rad, Hercules, CA, USA). The bands were quantified using the ImageJ software ( NIH, Rockville, MD, USA).

### 2.7 Mitochondria isolation and respiration measurements

The mitochondria were isolated from fresh gastrocnemius tissue with the Mitochondria Isolation Kit (ab288084, Abcam, Waltham, MA, USA), following the manufacturer's instructions. The oxygen consumption rate of isolated mitochondria was measured with a Seahorse Extracellular Flux Analyzer (Agilent, Santa Clara, CA, USA). The instrument was maintained at RT. The protein concentrations of isolated mitochondria preparations were measured by DC protein assay (BioRad, Hercules, CA, USA). Equal amounts of mitochondria (4 μg protein/well) were plated on the Seahorse cell culture microplate in 25 μL of Mitochondria Assay Solution (MAS). The assay medium was then added to the wells and incubated at 37 °C for 30 min and transferred to the analyzer for analysis. The respiratory stocks were loaded into the drug ports of a hydrated sensor cartridge in the following order: (A) ADP (4 mM final) (B) oligomycin (3 μM final), (C) FCCP (4 μM final), and (C) antimycin A (4 μM final) + rotenone (2 μM final). The tissue respiration assay protocol consisted of a minimum of three cycles of OCR measurements for each measurement period.

### 2.8 Histology and immunohistochemistry

The gastrocnemius tissues were fixed, sectioned, and stained using Hematoxylin and Eosin. Immunolocalization of Neutrophil Elastase (90120, Cell Signaling Technologies, MA, USA) was done using an HRP/DAB detection system. All the images were taken in Zeiss Axioscan 7 brightfield microscope.

### 2.9 Flow cytometry

The mitochondrial membrane potential was determined using TMRE staining. Briefly, 0.1 mg of isolated mitochondria from mice gastrocnemius muscle were incubated with TMRE (200 nM) for 15–30 min, and the fluorescence was measured using a flow cytometer at 488 nm excitation and 575 nm emission. Samples were measured using Attune NxT (ThermoFisher, MA, USA) and analyzed using FlowJo.10.8.1 (Tree Star, USA).

### 2.10 Statistical analysis

Data are presented as mean ± SEM. Statistical analysis was performed using GraphPad Prism 10.3.1 (GraphPad Software, La Jolla, CA, USA). The data was analyzed using ordinary ANOVA or t-test. *n* indicates the number of mice. *p* value < 0.05 was considered statistically significant. Significance (*p* value) is represented as ∗, where ∗ = ≤ 0.05, ∗∗ = ≤ 0.01, ∗∗∗ = ≤ 0.001, and ∗∗∗∗ = ≤ 0.0001.

## 3 Results

### 3.1 Physical activity in PB/PER-exposed mice after treatment with mitochondria-targeting agents

The exposure of mice to GWI agents affected their body weight and physical activity, as measured by hanging wire, rotarod and Noldus phenotyper tests [18,19]. After 10 days of PB/PER or DMSO exposure, mice were observed for 8 months to allow the development of GWI-like signs. After this period, the PB/PER-exposed animals were divided into four groups, one group served as vehicle control, the second group received metformin, the third group received MitoQ, and the fourth group received a combination of both. The effects on physical activity and endurance were measured using the hanging wire, rotarod and a phenotyper running wheel (Fig. 1). There was a sudden fall in the body weight of the mice during the PB/PER exposure regimen compared to the mice that received DMSO (vehicle) alone during this period (Fig. 1B). However, after eight months of observation, all PB/PER-treated mice exhibited a weight gain compared to the DMSO-treated group (Fig. 1C).

The muscle coordination and strength of mice in different treatment groups were assessed using the hanging wire test. We observed a significant drop in hanging time in mice exposed to PB/PER. The average time of hanging (sec) was 49.89 ± 6.88 in vehicle (DMSO) control, which was reduced to 32.54 ± 10.06 in PB/PER-exposed group and was restored to 39.24 ± 11.15 in Met/MitoQ combination-treated mice. Though the MitoQ-treated mice exhibited a significantly longer hanging time compared to the PB/PER group, this was not statistically significant from the group of mice that received either the combination therapy or metformin alone (Fig. 1D). As these experiments were performed in two batches, the results from each experiment were normalized to the DMSO group before combining the data and plotting the graph, so Y-axis does not show absolute values. The original data is provided in Supplementary Table S2. Next, to assess motor performance, rotarod technique was used. While latency to fall was reduced in PB/PER group (182.82 ± 32.02) compared to the control (247.36 ± 52.63), there was an improving trend in all the three treatment groups (Fig. 1E).

The distance traveled on the running wheel by the PB/PER exposed mice was less than in the vehicle (DMSO) control group but improved with MitoQ or the combination treatment (Fig. 1F). The counts in the running wheel were significantly reduced in the PB/PER group when compared to vehicle control or following treatments. Revolutions of the running wheel also decreased with PB/PER exposure and improved in treatment groups, though not significant. The mice treated with the combination formulation showed improvement in wheel zone entries, time spent, and distance covered on the running wheel compared to the PB/PER group. Shelter entries were significantly reduced in PB/PER group compared to the control which may be reflective of the reduced locomotor activity that was also evident in the total distance traveled and may indicate GWI like signs of fatigue and neuromuscular problems (Fig. 1F) (Table S2). The lack of treatment-related differences in the time spent in the shelter indicates a lack of anxiety-like behavior. Though some of the changes in these phenotyper parameters were not statistically significant, the trend was markedly visible suggesting the effect of PB/PER in developing GWI signs and the salutary effects of mitochondria targeted agents in muscle endurance.

### 3.2 GWI agent-induced tissue inflammation and oxidative stress were attenuated by metformin and MitoQ treatment

**Fig. 2:**
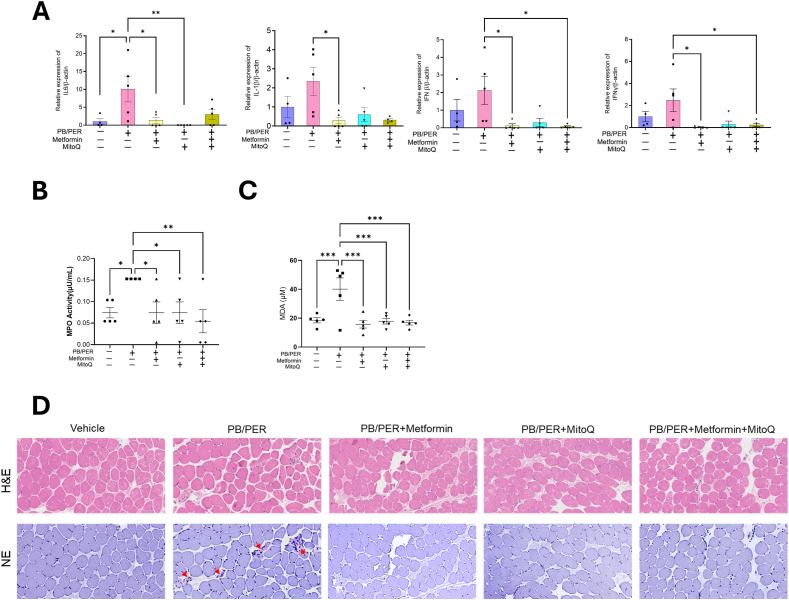
**Metformin and MitoQ reduce the inflammation and oxidative stress inthegastrocnemius muscle tissues of PB/PER exposed mice. (A)** mRNA levels of IL6, IL-1β, IFNβ, and IFNγ in the gastrocnemius tissue of mice exposed to PB/PER and treated with Veh,metformin, and/or MitoQ. The data were normalized to β-actin. The graphs are shown as individual data points along with Mean ± SEM. n = 4–5. One-way ANOVA test was used for statistical analysis. ∗p < 0.05,∗∗p < 0.01. **(B)and(C)** The effect of metformin, MitoQ, and their combination on MPO activity and MDA in the gastrocnemius tissue. The graphs are shown as individual data points along with Mean ± SEM, *n* = 4–5. Ordinary one-way ANOVA was used for analysis. ∗p < 0.05,∗∗p < 0.01,∗∗∗p < 0.001. **(D)** Representative images of H&E staining and immunohistochemical staining for neutrophil elastase (NE) with gastrocnemius tissues (continuous sections) of GWI mice (20X). Red arrows indicate elastase-positive neutrophils.

As skeletal muscle is a major tissue involved in fatigue, our subsequent experiments were focused on molecular changes in the gastrocnemius muscle, which is predominantly type 2 fiber. PB/PER exposure significantly increased the expression of proinflammatory cytokine genes, *IL-6*, IL-1β, IFNβ, and IFNγ, in the gastrocnemius muscle. The expression levels of all four cytokines were reduced in the GWI-induced mice treated with metformin, MitoQ, or the combination of metformin and MitoQ (Fig. 2A). Consistent with the GWI- associated exacerbated tissue inflammation, we also found enhanced oxidative stress in the muscle, as demonstrated by elevated myeloperoxidase activity (MPO) and malondialdehyde (MDA) levels in those exposed to PB/PER but not treated (Fig. 2B and C). The tissue levels of both MPO and MDA were restored when treated with the mito-targeting agents metformin, mitoQ, or the combination. We also observed sporadic elastase-positive neutrophil infiltration in sections of gastrocnemius muscle from untreated PB/PER exposed mice (Fig. 2D).

### 3.3 The effect of metformin and MitoQ on mitochondrial metabolism in GWI mice

**Fig. 3:**
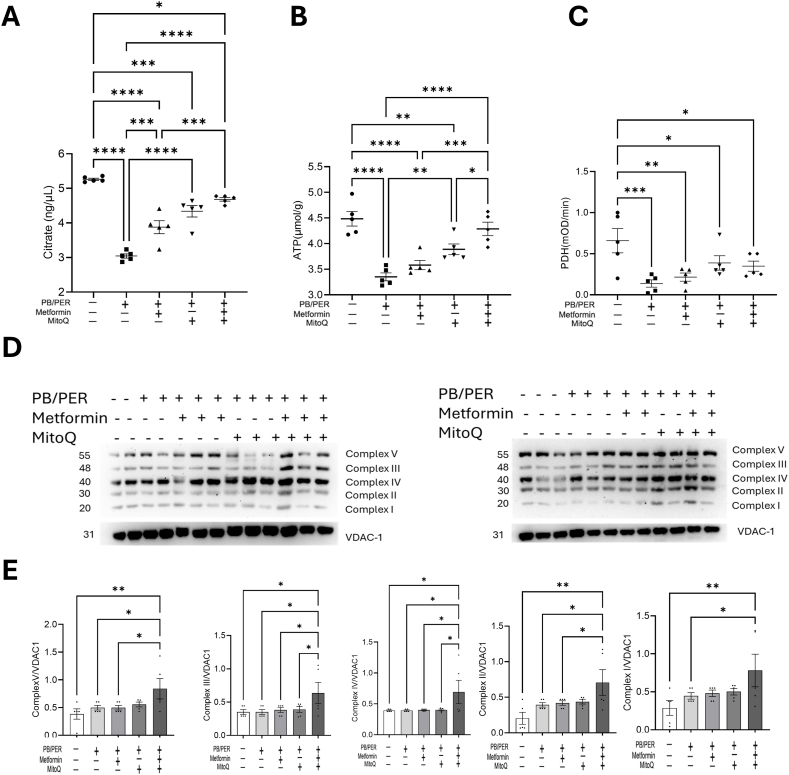
**Metformin and MitoQ improve mitochondrial metabolism in GWI mice. (A,B,C)** Metformin and MitoQ increase the citrate level, ATP level, and pyruvate dehydrogenase (PDH) enzyme activity in the gastrocnemius tissues of GWI mice. The graphs are shown as individual data points along with Mean± SEM. n=5. One-way ANOVA was used for analysis.∗p < 0.05,∗∗p < 0.01,∗∗∗p < 0.001,∗∗∗∗p < 0.0001. (**D,E)** Mitochondrial OXPHOS complexes in the gastrocnemius muscle of PB/PER administrated mice were detected by immunoblotting, n = 5 (n = 2–3 in each set). One-way ANOVA is used for statistical comparison between groups. The graphs are shown as individual data points along with Mean ± SEM.∗p < 0.05,∗∗p < 0.01.

Citrate is a key component of mitochondrial Krebs cycle. Our results showed a significant decline in tissue citrate levels in the gastrocnemius muscle of mice treated with PB/PER and vehicle (Fig. 3A). When PB/PER exposed mice were later treated with metformin, MitoQ or the combination, an elevated level of citrate was observed in the muscle, the largest increase was observed in the group of mice that received the combination of metformin and MitoQ (Fig. 3A). As expected, the ATP levels in gastrocnemius tissues of GWI mice showed a similar trend to that of total tissue citrate (Fig. 3B). This indicates a state of high energy availability in the metformin and MitoQ-treated groups compared to the mice in PB/PER-group. A reduced cellular energetics in the skeletal muscle with GWI agent exposure and its accentuation with metformin and MitoQ, were further established by demonstrating a decreased activity of pyruvate dehydrogenase that improved with the drug treatment (Fig. 3C). However, though the protein expression of mitochondrial ETC complexes were not significantly changed with PB/PER exposure, we observed elevated protein levels for these complexes in mice treated with metformin-MtioQ combination. The ETC enzymes tested include complex I: NADH ubiquinone oxidoreductase, complex II: succinate dehydrogenase, complex III: ubiquinol–cytochrome c oxidoreductase, complex IV: cytochrome *c* oxidase and complex V: ATP synthase (Fig. 3D and E).

### 3.4 Metformin and MitoQ improves mitochondrial respiration in GWI mice

**Fig. 4:**
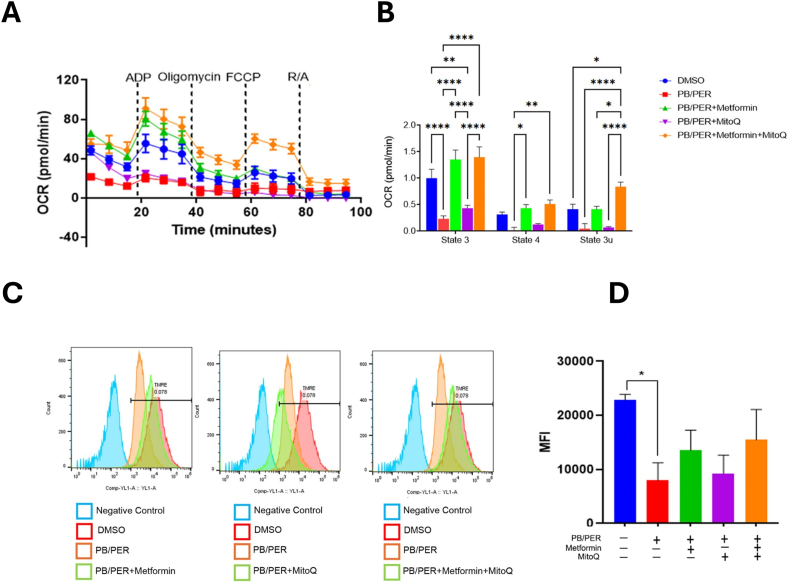
**Metformin and MitoQ restored the oxygen consumption rate (OCR) and mitochondrial membrane potential in GWI mice. (A, B)** Metformin and MitoQ treatment improved OCR in the mice's gastrocnemius tissue when compared to the untreated PB/PER-exposed mice. The assay was done using a Seahorse analyzer. R/A, rotenone+antimycin. n=3, One-way ANOVA was used for statistical comparison between different groups.∗p < 0.05,∗∗p < 0.01,∗∗∗p < 0.001,∗∗∗∗p < 0.0001 **(C, D).** The mitochondrial membrane potential was assessed by TMRE flow cytometry and analyzed by One-way ANOVA. MFI, mean fluorescence intensity. ∗p < 0.05.

Mitochondrial respiration was assessed by estimating the oxygen consumption rate (OCR) of isolated mitochondria under metabolic stress, using a Seahorse Extracellular Flux Analyzer. We observed a reduced basal OCR in the mitochondria isolated from the gastrocnemius muscle of PB/PER-treated mice, compared to the control. However, treatment with metformin, MitoQ, and the combination improved mitochondrial respiration. The combination of metformin and MitoQ improved the state 3U respiration more effectively than the individual drug treatments (Fig. 4A and B). A decrease in mitochondrial membrane potential is an indicator of mitochondrial dysfunction. Tetramethyl rhodamine ethyl ester perchlorate (TMRE) is a cationic dye that is readily sequestered by active mitochondria in response to their high membrane potential. Mitochondria that have lost membrane potential cannot accumulate TMRE. The exposure to GWI agents, PB and PER, decreased the mitochondrial membrane potential as shown by the reduction in TMRE fluorescence as assessed using flow cytometry. Whereas the combination of metformin and MitoQ as well as metformin alone improved the mitochondrial membrane potential. Surprisingly, mitochondria isolated from MitoQ treated mice did not show improved membrane potential (Fig. 4C and D). These data indicate that metformin or combination therapy can improve mitochondrial OCR and potential in mice exposed to PB/PER.

### 3.5 Metformin and MitoQ treatment rescue GWI-induced autophagy inhibition

**Fig. 5:**
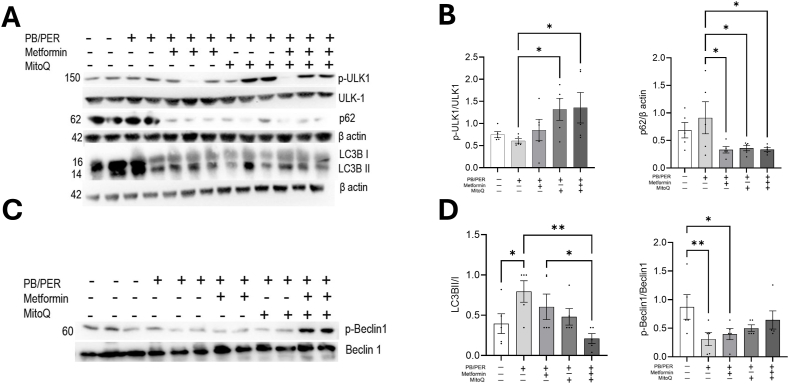
**Metformin and MitoQ activated autophagy. (A**–**D)** Metformin and MitoQ treatment increased the phosphorylation of ULK1, Beclin1 and decreased the expression of autophagy flux marker p62 and LC3BII/I ratio in comparison to the untreated PB/PER-exposed mice. Gastrocnemius tissue was immunoblotted against anti-phospho ULK1, ULK1, p62, LC3B, anti-phospho beclin1, and beclin1, and was detected by ECL. One-way ANOVA is used for statistical comparison. The graphs are shown as individual data points along with Mean ± SEM. n = 5.∗p < 0.05,∗∗p < 0.01.

To further test the mechanism of GWI agent induced chronic fatigue, we next examined the effect of GWI agent exposure on autophagy in the skeletal muscle. While autophagy was reduced with PB/PER exposure as observed by a reduction in *p*-ULK-1, treatment with metformin or MitoQ alone or in combination increased *p*-ULK-1 protein expression. The highest *p*-ULK-1 expression was observed in the group of mice that received the combination therapy. Autophagy is an essential process for removing damaged cell organelles, including mitochondria. The immunoblot results show the reduced expression of *p*-ULK1 and increased accumulation of the autophagy flux marker, p62, in the gastrocnemius tissues of PB/PER exposed mice. The increased p62 expression indicates the accumulation of autophagy substrate when exposed to GWI agents. An increase in the ratio of LC3B-II/I together with p62 shows inhibition of autophagosome degradation in the PB/PER group (Fig. 5A and B). ULK1 directly phosphorylates beclin1 which is an important constituent of the VSP34 complex [23]. *p*-Beclin 1 levels are reduced in the muscle of PB/PER treated mice indicating a decrease in autophagic process with PB/PER (Fig. 5C and D). Metformin and MitoQ treatment were found to improve the GWI agent-induced inhibition of autophagy, thereby restoring the cell's innate recycling machinery.

### 3.6 Metformin and MitoQ activate AMPK and mitochondrial biogenesis in GWI mice

**Fig. 6:**
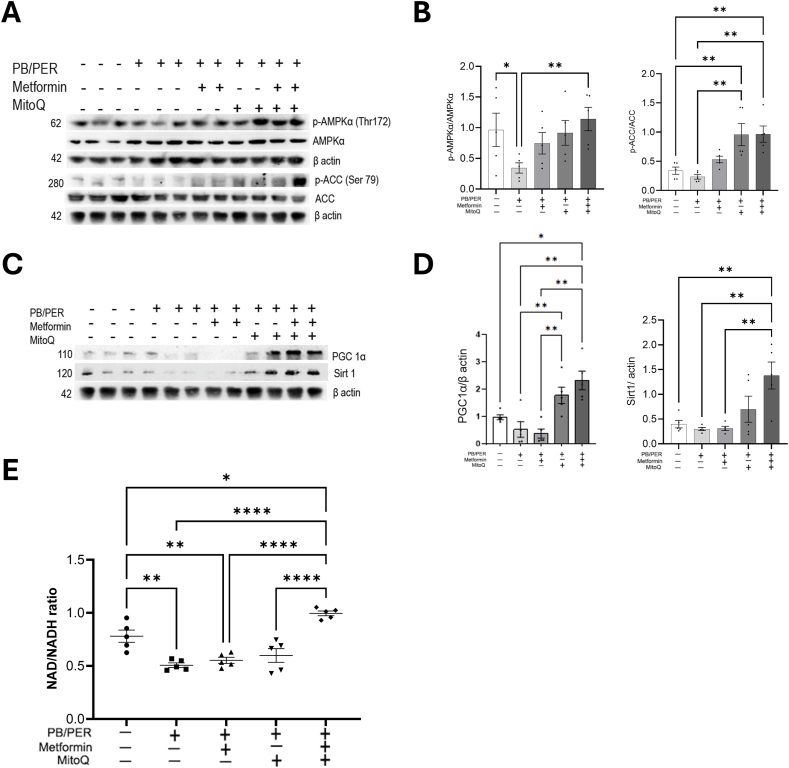
**Metformin and MitoQ activated AMPK**α**, Pgc1α and Sirt1. (A**–**D)** Metformin and MitoQ treatment increased the phosphorylation of AMPKα at threonine 172 residue, ACC at serine 79 residue, Pgc1α and Sirt1 compared to the untreated PB/PER-exposed mice. Gastrocnemius tissue was immunoblotted using specific antibodies and detected by ECL. The graphs are shown as individual data points along with Mean ± SEM, n = 4–5. One-way ANOVA was used for statistical comparison.∗p < 0.05,∗∗p < 0.01. **(E)** NAD/NADH ratio was determined as described in the Materials and Methods. The graphs are shown as individual data points along with Mean ± SEM, n = 5. One-way ANOVA was used for statistical comparison. ∗p < 0.05,∗∗p < 0.01,∗∗∗p < 0.001,∗∗∗∗p < 0.0001.

Pgc1 alpha co-activates and coordinates the expression of genes involved in mitochondrial biogenesis and oxidative phosphorylation. Activation of Pgc-1α by AMPK and deacetylation by Sirt1 are pre-requisites for its activation. AMPK is an energy sensor. As shown in Fig. 6A, we found reduced phosphorylation levels of AMPKα in the gastrocnemius muscle of mice treated with PB/PER and veh, whereas AMPK phosphorylation improved with metformin, MitoQ, and the combination treatment, with the combined drug showing a synergistic effect. These drugs also phosphorylated the Serine 79 residue of ACC, the downstream target of AMPKα (Fig. 6A and B). Metformin and MitoQ treatment were also found to increase the expression of Pgc1α and Sirt1. Sirt1 uses nicotinamide adenine dinucleotide (NAD+) as a cofactor and deacetylates Pgc-1α to promote mitochondrial biogenesis and maintain mitochondrial function. NAD/NADH ratio showed an increase in NAD levels when the mice were treated with both metformin and MitoQ, which correlates with the enhanced Sirt1 activity in the combination treatment (Fig. 6C-E).

### 3.7 Metformin and MitoQ treatment activate/restore the antioxidant mechanism in GWI mice

**Fig. 7:**
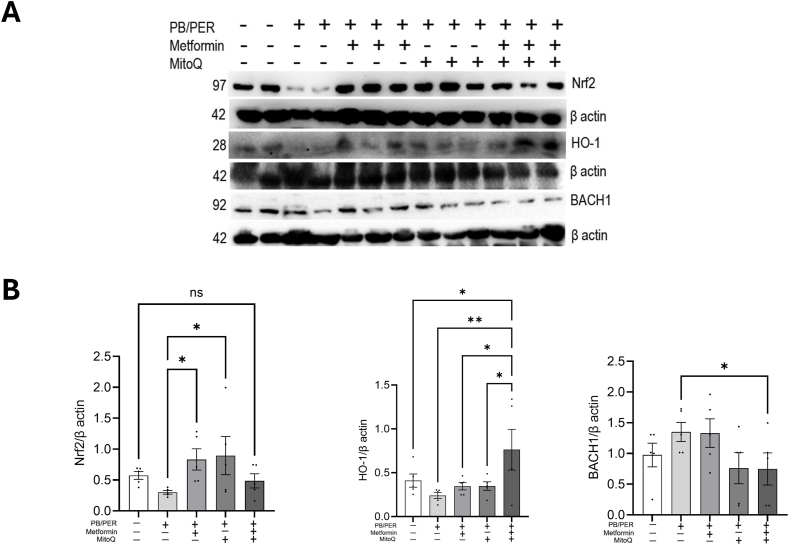
**The effect of** m**etformin and MitoQ on Nrf2, HO-1, and Bach1 levels in mice exposed to PB/PER. (A**–B**)** The gastrocnemius tissues were immunoblotted using antibodies to Nrf2, HO-1, BACH1 and detected by ECL. n = 4–5. One-way ANOVA was used for statistical comparison.∗p < 0.05,∗∗p < 0.01, ns=non significant

The treatment of GWI mice with mitochondrial targeting agents metformin and MitoQ increased levels of Nrf2, which promotes the expression of antioxidant gene, HO-1 (Fig. 7A and B). Activation of HO-1 has been shown to break down heme to produce carbon monoxide, iron, biliverdin, and bilirubin. While we observed a reduction in Nrf2 levels in the muscle of mice treated with PB/PER, metformin and MitoQ significantly increased Nrf2 protein levels compared to the PB/PER group. Though HO-1 showed an increasing trend, it was most elevated in mice that received the combination therapy. As shown in Fig. 7B, there was an inverse relationship between Nrf2 levels and Bach1 in the untreated PB/PER-exposed group. Bach 1 is a transcriptional repressor, which competes with Nrf2 for binding to the ARE (antioxidant response elements). The Nrf2-HO-1-Bach1 pathway is a crucial mechanism for cellular defense against oxidative stress.

## 4 Discussion

The mouse model of GWI was developed as described in Materials and Methods section, by intraperitoneal injection of 0.7 mg/kg of PB and 200 mg/kg of PER daily for 10 consecutive days and observing for 8 months [17,19,24]. Prior studies have shown that animals exposed to GWI agents like PB, PER, and DEET alone or in combination exhibited cognitive impairment, oxidative stress, inflammation and fatigue [[25], [26], [27], [28]]. We observed a sudden decline in the body weight of the mice during exposure to PB/PER. But after eight months of observation there was an increase in body weight in PB/PER-treated groups, compared to the control group, consistent with the report that majority of Gulf War Veterans are overweight [[29], [48], [49]]. This is the first study that compared the effects of MitoQ, metformin and the combination formulation in a GWI mouse model. We used MitoQ, instead of CoQ10 as it is superior to CoQ10. CoQ10 has poor absorption, and its ability to accumulate in the mitochondria is limited by high lipophilicity, large molecular weight and poor aqueous solubility [30]. However, MitoQ is a lipophilic mitochondria-targeting triphenyl phosphonium cation conjugated to coenzyme Q10 to facilitate accumulation within the mitochondria [31]. It has been known to block ROS and reduce/prevent mitochondrial oxidative damage. MitoQ is also reported to activate the Nrf2-mediated antioxidant pathway to protect against mitochondrial damage [32]. Studies have shown beneficial effects of MitoQ treatment in various conditions such as heart failure, cardiac hypertrophy, liver fibrosis, I/R injury and neurodegenerative diseases [12,33,34].

Some studies have previously reported the loss of skeletal muscle strength and reduced physical activity in GWI mice models [35,36]. However, targeting mitochondria to ameliorate fatigue has not been investigated. We investigated grip strength, running wheel performance, and physical activity assessed by hanging wire, rotarod, and PhenoTyper tests (Fig. 1D–F). We further determined the changes in skeletal muscle mitochondrial activity in response to mitochondria targeted treatments. Several studies have implicated neuroinflammation as a causative factor for GWI symptoms. A recent study found that a combination of ocular coherence tomography measures and plasma inflammatory cytokine profile predicted GWI symptoms with good sensitivity (83%) though specificity was low [37]. A phase 2 randomized controlled trial using low dose prednisone showed improvement in physical health-related quality of life suggesting inflammation is a critical factor in GWI symptoms [38]. PB treatment alone or in combination with stress was found to elicit dysregulation of peripheral immune responses, and suggested that PB dysregulates the cholinergic anti-inflammatory pathway, resulting in the onset of neuroinflammation. We observed an increase in tissue proinflammatory cytokines in the gastrocnemius muscle of mice treated with PB/PER and a restoration after treatment with metformin, MitoQ or Met + MitoQ. The accentuated tissue expression of proinflammatory cytokines could be contributing to the chronic fatigue observed in GWI. This observation of significant inflammation in skeletal muscle of mice with GWI signs is novel as most of the investigations on inflammation observed with GWI are focused on brain and circulation.

We used a battery of voluntary running wheel and additional home cage behaviors to assess the effects of PB/PER exposure as well as the treatments on fatigue. This was accomplished by placing individual mice in Noldus PhenoTyper® home-cage monitoring systems and video-tracking a variety of behaviors using EthoVision XT® software over an 18-h period. Each of the modules was equipped with a voluntary running wheel, a lickometer to measure drinking behavior, and an IR-translucent shelter (10cm×10cm). The tracking system was thus set up to measure motivation to exercise and fatigue (running wheel activity), open-field exploratory behavior in all areas of the arena, excluding the location of the voluntary running wheel and shelter, and to measure entries and time spent in the shelter, as well as on the running wheel. As an example, animals exhibiting anhedonia and or/anxiety-like behaviors would be expected to travel less distance in the open field, show a lower number of licks to drink water on the lickometer and a reduced number of wheel zone entries. They would also be expected to spend more time in the sheltered zone, which is designed to provide a safe enclosure from the open field portion of the arena. Separately, we also assessed neuromotor strength using a hanging wire and a rotarod. The in vivo exposure of mice to PB/PER reduced the distance traveled, the number of revolutions in the running wheel, running wheel counts, and shelter entries in the PhenoTyper, indicating the neuromuscular impact of GWI agents on the overall activity of the mouse. Most of these parameters were reversed with either metformin, MitoQ or Met/MitoQ combination treatment. Neuromotor endurance was also similarly decreased with PB/PER, as shown by the reduced hanging time and the latency to fall from the rotarod. These results confirm the induction of GWI-like behavior in mice and demonstrate that mitochondrial potentiation is a feasible method for treating GWI.

Both metformin and MitoQ are known to modify mitochondrial functions. Therefore, we measured several metabolic markers related to mitochondrial function in the gastrocnemius muscle of PB/PER administered animals treated with metformin, MitoQ or Met + MitoQ. Citrate is a key Krebs cycle intermediate. While it is an important substrate in energy metabolism in the mitochondria, it is also transported to the cytosol for the generation of cytosolic acetyl-CoA. The significant reduction in citrate levels following PB/PER exposure suggests an energetic deficiency in the skeletal muscle in the GWI mice, which was mostly restored by the combination therapy, though metformin and MitoQ individually also had a salutary effect. Consistent with the citrate levels, total tissue ATP was also significantly reduced in the GWI mice, while the metformin, MitoQ, and Met/MitoQ combination had progressively better effects in normalizing the ATP levels. The relationship between ATP levels in the gastrocnemius muscle and its function can be easily understood from previous reports that suggest a 50 % reduction of ATP content with aging, confirming that GWI agents may effectively cause muscle fatigue [39]**.** This energetics dependent fatigue phenotype was further ascertained by assaying the activity of pyruvate dehydrogenase (PDH), the gatekeeper of mitochondria, which is the key enzyme that catalyzes the conversion of pyruvate to acetyl CoA [40]. The group of mice administered PB/PER and treated with Veh showed the least activity of PDH, while those treated with the combination of metformin and MitoQ restored the activity. From these experiments alone it is not possible to conclude whether reduced activity of PDH alone is causing mitochondrial function decline nor the cause of a decrease in PDH activity, because PDH activity depends on several factors such as activation of pyruvate dehydrogenase kinase (PDK) which is an inhibitor of PDH, change in substrate utilization, and hypoxic conditions. Nevertheless, reduced PDH activity has the potential to reduce the acetyl-CoA influx into the citric acid cycle. More mechanistic studies are required to understand the effect of PB/PER on PDH activity. Our experiments designed to test for a quantitative decline in the levels of each of the complexes involved in the electron transport chain (ETC), did not yield any notable changes in the untreated PB/PER group, suggesting that PB/PER had no effect on protein levels of these complexes.

We next asked whether the measures of molecular markers of mitochondrial function, such as citrate levels and PDH activity, mirror mitochondrial respiration. Towards this, we tested the respiration of mitochondria isolated from the gastrocnemius muscles of mice treated with vehicle or drugs after PB/PER exposure. We observed reduced OCR and ADP-induced respiration in mice exposed to PB/PER but not treated with the drugs. There was a significant reduction in State 3, State 4 and State 3u respiration. However, both drugs individually and in combination improved State 3, State 4 and State 3U respiration. This study indicates a functional decline of mitochondria with 10.13039/100010264PB/PER exposure and provides supporting evidence to show that the two 10.13039/501100008602GWI agents have adverse effects on skeletal muscle mitochondrial function, which can be reversed with mitochondria targeting agents, metformin and MitoQ. We also found significantly reduced mitochondrial membrane potential in mice administered PB/PER that was at least partially restored by the Met-/MitoQ combination. This experiment provides additional evidence for the deleterious effect of PB/PER on mitochondria and demonstrates that by targeting mitochondria, skeletal muscle energy supply can be improved. Previous studies on GWI veterans also have demonstrated mitochondrial functional decline [10,41]. Metformin and MitoQ are widely used in animal studies and metformin has been extensively used in human [42,43].

The energetic decline in the skeletal muscle with PB/PER and improvement after Met/MitoQ treatment are also evident from the increased phosphorylation of AMPK at Thr 172 as well as modulation of Sirt1/Pgc-1a axis. These results indicate that one possible pathway by which metformin improved mitochondrial function was by activating AMPK and Sirt1, the activity of these converges on Pgc1-a which is a mitochondrial biogenesis factor. Activated AMPK phosphorylates Pgc-1a while Sirt1 deacetylates Pgc-1a. Increased AMPK leads to higher NAD levels, the latter being a substrate of Sirt1 and increasing its deacetylase activity. The phosphorylation, and deacetylation are necessary conditions for activating Pgc-1a. The functional status of AMPK was further confirmed by testing ACC (acetyl CoA carboxylase) phosphorylation as ACC is a direct target of phosphorylated AMPK. *p*-ACC increases the beta oxidation of fatty acids to yield acetyl CoA, the substrate of TCA cycle. The interrelationship between Sirt1 and AMPK and their role in energy sensing and mitochondrial function are well reported and our studies show their possible role in the pathology associated with GWI.

**Fig. 8:**
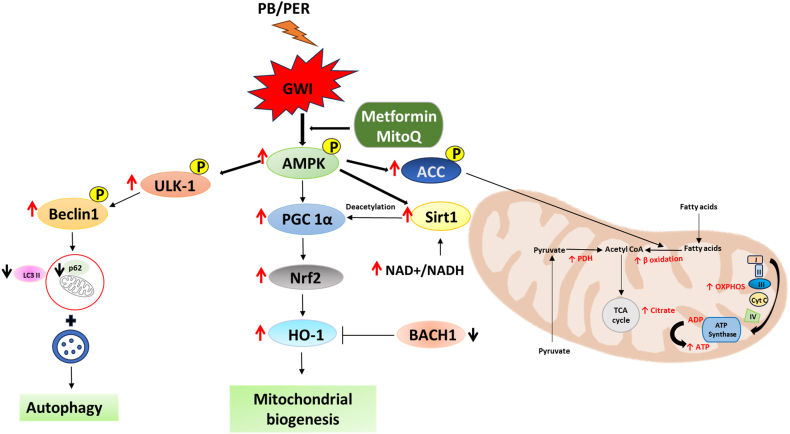
**Metformin and MitoQ activate AMPK signaling, antioxidant response, and improve mitochondrial function in mice exhibiting signs of GWI after PB/PER exposure.** Metformin and MitoQ treatment improved mitochondrial biogenesis possibly through AMPK/Sirt1/Pgc1α signaling axis and mitophagy through AMPK/ULK1 axis to restore the metabolic homeostasis in PB/PER exposed mice.

The oxidative stress observed as measured by elevated MPO activity and the mitochondrial functional decline seen in PB/PER treated mice are interrelated physiological responses, and MitoQ has been shown to have therapeutic effects in such conditions. Metformin and MitoQ prevented the loss of Nrf2 by PB/PER. Nrf2 activates its target gene HO-1, an antioxidant that breaks down heme. The products of heme catabolism exert antioxidant and anti-inflammatory effects, thus protecting the cell from oxidative damage [44]. We found a significant increase in HO-1 protein levels only in response to the combination drug, so the impact of HO-1 on Bach1 expression seems to be only in the Met/MitoQ combination group. Nevertheless, targeting the Nrf2-HO-1 axis, either by activating Nrf2 or inhibiting Bach1 is a promising therapeutic strategy for various diseases caused by oxidative stress damage (Fig. 8).

The bioenergetic impairment following the exposure to PB/PER can initiate cellular stress [45]. However, these animals did not show elevated expression of commonly used markers of senescence in multiple tissues tested (Supplemental Fig. 1). The stress or damage-induced intracellular organelles are normally cleared by autophagy, a homeostatic process. The recycling removes damaged organelles and generates metabolic substrates to maintain cellular homeostasis. However, a reduced autophagic process will lead to accumulation of oxidative products and intracellular debris leading to cell death. In mice exposed to PB/PER, we show a decrease in the autophagy process as demonstrated by reduced *p*-ULK1 and *p*-Beclin 1, and increased p62 and LC3BII, and an increased autophagy when these mice were treated with Met/MitoQ. The increase in autophagy was more prominent in mice that received the combination dose. The change in phospho-ULK1 levels observed was consistent with the changes in *p*-AMPK. Activated ULK1 (*p*-ULK1) is an initiator of autophagy, which phosphorylates Beclin-1, a main component of the class III phosphatidylinositol 3-kinase (PI3K-III) complex, which is essential for autophagy [46]. It is to be noted that AMPK interacts with and phosphorylates ULK1 to promote autophagy [47]. While AMPK activation is expected with energy reduction, consistent chronic deprivation could be the reason for reduced pAMPK observed with PB/PER exposure (Fig. 8).

In this model, the animals were exposed to PB/PER by intraperitoneal administration for 10 days and treated with Veh or Met/MitoQ before assessing behavioral and molecular changes. Others had tested these animals immediately after exposure, but did not find any detectable GWI symptoms [17,19]**.** This indicates that PB/PER exposure induces subtle molecular damage, which is perpetuated and amplified gradually over a period of months and possibly maintained for life. Our experiments with metformin and MitoQ formulations show that we can break this vicious cycle even after the manifestation of GWI symptoms, by proper intervention, specifically that target mitochondria. However, additional experiments are needed to determine whether limited time treatment is sufficient to cure the symptoms, or if they will relapse after the short-term treatment ceases. The treatment regimen in this study was every other day for eight weeks.

In conclusion, our results demonstrate significant impairments in gastrocnemius muscle function and mitochondrial activity, accentuated tissue inflammation, increased oxidative stress and reduced autophagy in mice intraperitoneally exposed to PB/PER. Our study further shows that appropriately targeting host mitochondria can have a therapeutic effect. The two drugs we used, metformin and MitoQ are well-known mitochondria modulators, and our results show that a combination of metformin and MitoQ is superior to either of them alone in reducing molecular markers of GWI.

## CRediT authorship contribution statement

**Lun Cai:** Writing – review & editing, Writing – original draft, Methodology, Formal analysis, Data curation. **Mundanattu Swetha:** Writing – review & editing, Writing – original draft, Methodology, Investigation, Formal analysis, Data curation. **Abraham Raji:** Writing – review & editing, Writing – original draft, Methodology, Data curation. **Alvin V. Terry:** Writing – review & editing, Writing – original draft, Methodology, Funding acquisition, Conceptualization. **Raghavan Pillai Raju:** Writing – review & editing, Writing – original draft, Supervision, Resources, Project administration, Investigation, Funding acquisition, Data curation, Conceptualization.

## Funding information

This work was mainly supported by the grant W81XWH-21-1-0244 from the 10.13039/100000005United States Department of Defense and partially by the grant I01BX006256 from the US Department of Veterans Administration, and also some funds from 10.13039/100012127Augusta University.

## Declaration of competing interest

The authors declare that they have no known competing financial interests or personal relationships that could have appeared to influence the work reported in this paper.

## Data Availability

Data will be made available on request.
