# Supplementary material for: Mitochondria-targeted therapy with metformin and MitoQ reduces oxidative stress, improves mitochondrial function, and restores metabolic homeostasis in a murine model of Gulf War Illness
Source: Redox Biol. 2025 Jun 4;85:103714. doi: 10.1016/j.redox.2025.103714 (PMC12221886; doi:10.1016/j.redox.2025.103714)
Supplement: Multimedia component 1 [file mmc1.pdf]

Supplementary Data

Table S1. Mouse primer sequences used

Sl. No.	Gene	**Forward primer sequence (5'-3')**	**Reverse primer sequence (5'-3')**
1	IFNβ	CCAGCTCCAAGAAAGGACGA	CGCCCTGTAGGTGAGGTTGAT
2	IFNγ	CCCTATGGAGATGACGGAGA	ACCCAGTGCTGGAGAAATTG
3	IL6	TGGAGTCACAGAAGGAGTGGCTAAG	TCTGACCACAGTGAGGAATGTCCAC
4	IL-1β	AACCTGCTGGTGTGTGACGTTC	CAGCACGAGGCTTTTTTGTTGT
5	p16ink4a/Cdkn2a	CCCAACGCCCCGAACT	GCAGAAGAGCTGCTACGTGAA
6	p21/Cdkn1a	GTTCCGCACAGGAGCAAAGT	ACGGCGCAACTGCTCAC
7	β-actin	CGCCACCACTTCGCCATGGA	TACAGCCCGGGGAGCATG

Table S2. Summary of Phenotyper, hanging wire and rotarod tests of GWI mice, n=49.

**Group**		**Time of hanging (sec)**	**Rotarod Latency to fall (sec)**	**Distance Traveled (cm)**	**Running Wheel (counts)**	**Running Wheel (Revolutions)**	**Wheel Zone Entries**	**Time spent on Running Wheel (sec)**	**Distance on Running Wheel (cm)**	**Lickometer (number of licks)**	**Shelter Entries**	**Time in Shelter (sec)**
Vehicle	Average	49.89167	247.3633	17217.99	37.33889	745.9639	59.38333	1270.968	327.9257	155.275	40.575	4087.583
	SEM	6.877667	52.63667	1527.674806	3.838889	56.54722	14.77222	26.80761	24.85816	5.636111	1.619444	306.8834
PB/PER	Average	32.5465	182.8267	13858.98	28.675	536.475	45.70556	1054.429	235.8344	139.1444	26.06111	4121.222
	SEM	10.0625	32.02667	1016.0665	2.369444	66.83611	8.65	67.36721	29.38115	18.36667	4.894444	498.9171
PB/PER + Metformin	Average	41.13375	219.79	15333.27	37.40278	617.4444	80.52778	1193.085	271.4286	189.8611	33.61111	4085.355
	SEM	4.997708	4.11	229.1177778	0.569444	31.5	8.25	16.01986	13.8474	12.13889	1.444444	4.017833
PB/PER + MitoQ	Average	54.843	227.7867	17377.66	42.62222	776.6694	73.45556	1382.81	341.4239	186.8167	34.80833	3852.377
	SEM	10.83767	0.246667	2759.727056	5.066667	120.7528	9.9	197.9922	53.08292	18.35	0.836111	631.2811
PB/PER + Metformin+ MitoQ	Average	39.23633	224.4367	17736.44	40.47222	729.5611	69.25	1409.899	320.7151	166.2	34.51944	3829.142
	SEM	11.15233	3.556667	471.2690278	0.472222	29.71667	6.805556	99.79569	13.06345	7.8	4.119444	24.70514

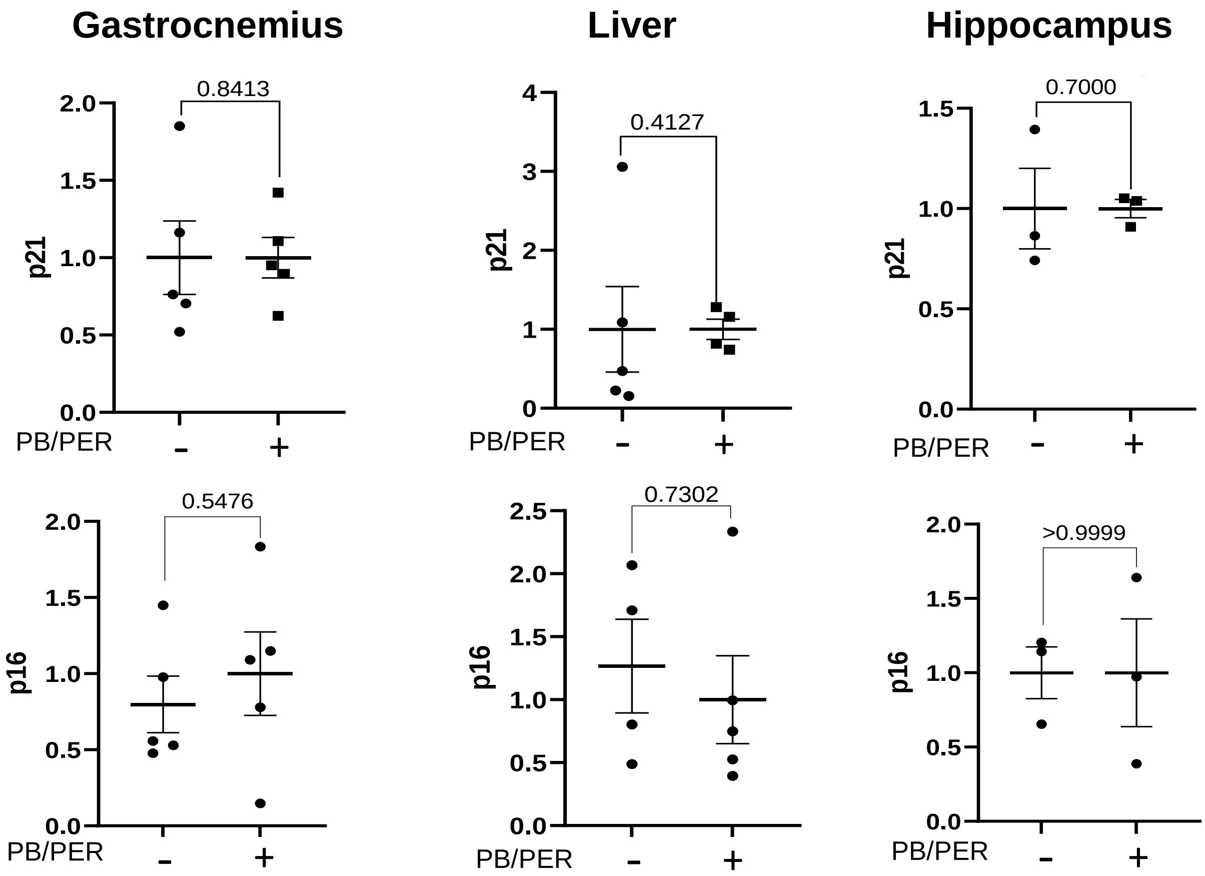

Figure S1. PB/PER exposure did not increase the gene expression of senescence markers, p21 and p16 in GWI mice. Relative expression of mRNA levels of cdkn1a (p21) and cdkn2a (p16) in gastrocnemius, liver, and hippocampus tissues of mice received DMSO (Vehicle) or PB/PER. The data was normalized to β-actin. The graphs are shown as individual data points along with Mean ± SEM. n=3-5. Mann Whitney non-parametric test was used to calculate p value.
